# Acceptability of telephone-based pain coping skills training among African Americans with osteoarthritis enrolled in a randomized controlled trial: a mixed methods analysis

**DOI:** 10.1186/s12891-020-03578-7

**Published:** 2020-08-14

**Authors:** Chamara J. Dharmasri, Ida Griesemer, Liubov Arbeeva, Lisa C. Campbell, Crystal W. Cené, Francis J. Keefe, Eugene Z. Oddone, Tamara J. Somers, Kelli D. Allen

**Affiliations:** 1 Department of Medicine, University of North Carolina, Chapel Hill, NC USA; 2 Department of Health Behavior, University of North Carolina, Chapel Hill, NC USA; 3 Thurston Arthritis Research Center, University of North Carolina, 3300 Thurston Bldg, CB# 7280, Chapel Hill, NC 27599 USA; 4 Department of Psychology, East Carolina University, Greenville, NC USA; 5 Department of Psychiatry and Behavioral Science, Duke University, Durham, NC USA; 6 Center of Innovation to Accelerate Discovery and Practice Transformation, Durham VA Health System, Durham, NC USA; 7 Department of Medicine, Duke University Medical Center, Durham, NC USA

**Keywords:** Osteoarthritis, Pain, Coping, Acceptability, Disparities, Mixed methods

## Abstract

**Background:**

Osteoarthritis (OA) disproportionately impacts African Americans compared to Caucasians, including greater pain severity. The Pain Coping Skills Training for African Americans with Osteoarthritis (STAART) study examined a culturally enhanced Pain Coping Skills Training (CST) program among African Americans with OA. This mixed methods study evaluated the acceptability of the Pain CST program among STAART participants.

**Methods:**

STAART was a randomized controlled trial evaluating the effectiveness of an 11-session, telephone-based pain CST program, compared to a usual care control group. Participants were from the University of North Carolina and Durham Veterans Affairs Healthcare Systems. The present analyses included 93 participants in the CST group who completed a questionnaire about experiences with the program. Descriptive statistics of the questionnaire responses were calculated using SAS software. Thematic analysis was applied to open-response data using Dedoose software.

**Results:**

Participants’ mean rating of overall helpfulness of the pain CST program for managing arthritis symptoms was 8.0 (*SD* = 2.2) on a scale of 0–10. A majority of participants reported the program made a positive difference in their experience with arthritis (83.1%). Mean ratings of helpfulness of the specific skills ranged from 7.7 to 8.8 (all scales 0–10). Qualitative analysis of the open-response data identified four prominent themes: Improved Pain Coping, Mood and Emotional Benefits, Improved Physical Functioning, and experiences related to Intervention Delivery.

**Conclusions:**

The high ratings of helpfulness demonstrate acceptability of this culturally enhanced pain CST program by African Americans with OA. Increasing access to cognitive-behavioral therapy-based programs may be a promising strategy to address racial disparities in OA-related pain and associated outcomes.

**Trial registration:**

NCT02560922, registered September 25, 2015.

## Background

Osteoarthritis (OA) is a leading cause of pain and disability in the United States [1]. The annual prevalence of arthritis, including OA, is projected to increase to over 78 million by 2040 [2]. African Americans experience greater prevalence of OA than Caucasians, as well as more severe OA-related pain and functional limitations [3–7]. For example, a U.S. study using data from the National Health and Nutrition Examination Survey reportedthe point prevalence of radiographic knee osteoarthritis was 52% among Black adults and 36% among White adults [7]. Studies also indicate that African Americans report greater levels of pain catastrophizing and that racial differences in pain coping patterns may be key contributors to disparities in pain severity [5, 6, 8, 9]. Pain Coping Skills Training (CST) has been effective in helping patients with OA, especially those who report greater pain catastrophizing, more maladaptive coping strategies, and overall lower ability to cope and control pain [10–12]. Therefore, Pain CST may be an important strategy for improving pain-related outcomes among African Americans with OA.

The Pain Coping Skills Training for African Americans with Osteoarthritis (STAART) study adapted a pain CST program based on input from African Americans with OA regarding cultural factors (e.g., religion, spiritual beliefs, lived experiences as African Americans) relevant to coping behaviors [13]. Following this adaptation, a randomized controlled trial examined the effectiveness of the culturally-enhanced pain CST program, delivered via telephone over 3 months, among African Americans with OA. Participants who received the pain CST intervention had significant improvements in pain coping and arthritis self-efficacy, immediately following program completion, compared with a waitlist control group [14].

In addition to evaluating the effectiveness of the pain CST program, it is important to obtain participants’ feedback on their perceptions of the program, as this is a patient-centered approach and captures information beyond standardized outcome measures. Accordingly, the objective of this secondary analysis of the STAART study was to report on participants’ responses to questions regarding their experiences and perceptions of the program, using a combination of quantitative and qualitative methods.

## Methods

### STAART study overview and participants

The STAART Study was a randomized controlled trial of 248 African Americans with symptomatic hip or knee OA [13, 14]. Half of the participants were recruited from the University of North Carolina (UNC) Healthcare System and half were from the Durham Veteran Healthcare System (DVAHCS). Three recruitment methods were utilized. The primary method involved identifying African American patients with OA diagnosis codes at UNC and DVAHCS, mailing a letter inviting participation, and following up with a telephone call. Other methods included advertisements at the study sites and surrounding communities to facilitate self-referral and allowing providers to give study pamphlets to patients or directly refer patients to the study team with their permission. Inclusion criteria for the STAART Study were: 1) Self-reported diagnosis of knee or hip OA from a medical professional, 2) Self-report of pain, aching, or stiffness in one or both knees or hips on most days of the week, 3) Patient of UNC or DVAHCS. Exclusion criteria have been described previously [13, 14].

STAART participants were randomized equally to pain CST and waitlist control groups in a parallel-group design with blinded researchers [13, 14]; these analyses include only the Pain CST group participants. Detailed methods have been published previously, describing the sampling strategy and intervention details [13, 14]. The Pain CST intervention involved 11 phone-based sessions, which were delivered by counselors with experience in psychological interventions; training and fidelity checks of the counselors have been described previously [13]. Sessions were conducted approximately weekly and lasted about 30–45 min. Topics for each session are described in Table 1. Participants were given handouts and an audio recording to support progressive muscle relaxation. During each session, a counselor instructed participants in a new cognitive and behavioral pain coping skill, guided participants in rehearsal of the skill (when appropriate), gave instructions for home-based practice of the skill and reviewed participants’ home practice from the prior session. Participants gave informed consent prior to being enrolled in the study. Study procedures were approved by the UNC and DVAHCS Institutional Review Boards. This study adhered to CONSORT guidelines for randomized trials [15].

**Table 1:** Pain Coping Skills Training Session Topics

Session 1	Introduction, Rationale for Pain Coping Skills, Progressive Muscle Relaxation
Session 2	Mini Relaxation Practices and Communicating with Significant Others about Pain and Coping Mini-Relaxation Practices
Session 3 & 4	Managing Unhelpful Mood
Session 5	Activity Pacing
Session 6	Pleasant Activities
Session 7	Pleasant Imagery and Other Distraction Techniques
Session 8	Physical Activity and Osteoarthritis
Session 9	Weight Management and Osteoarthritis
Session 10	Skills Review and Problem Solving
Session 11	Relapse Prevention and Maintenance Relapse Prevention

### Participant baseline characteristics

The following participant demographic and clinical characteristics were assessed via self-report at baseline, prior to randomization: age, sex, ethnicity (Hispanic/Latino descent or not), education level (categorized as above high school education vs. not), work status (currently working vs. not), household financial state (able meet basic expenses with a little left over for extras vs. not), marital status (married/living with a partner as married vs. not), Western Ontario and McMaster Universities Osteoarthritis Index (WOMAC; measure of lower extremity pain, stiffness and function) [16], Coping Strategies Questionnaire - Total Coping Attempts score (includes domains of Diverting Attention, Ignoring Sensations, Coping Self-Statements, Reinterpreting Pain Sensations, Praying-Hoping, and Increasing Behavioral Activities) [17, 18], Pain Catastrophizing Scale [19], and total number of comorbidities [20].

### Participant feedback questionnaire

At 3-month follow-up, shortly after completing the pain CST intervention, participants were asked a series of questions by an unblinded study team member, via telephone, to elicit their feedback on the intervention. The study coordinator typically administered the feedback questionnaires, but in some cases the coordinator was not available, and the study counselor administered the questionnaire. These questions included both closed and open-ended questions, as shown in Table 2. General topics included perceptions of helpfulness of the program and the skills taught, descriptions of changes in personal experiences and abilities, and feedback on advantages or potential barriers. Questions 1 and 2 focused on overall feedback, while Question 3 was session-specific. About 3 months after follow-up assessments began, Question 3 was modified to gather more targeted responses regarding feedback. We did not include the responses to Question 3 for the subset of 10 participants who completed the earlier version of this question.

**Table 2:** Patient Intervention Feedback Questions

1) On a scale of 0–10, with 0 being not helpful at all and 10 being very helpful, how much did this program help you to manage your arthritis symptoms? a) What is the reason you picked a “(score from #1)”?	
2) Has participating in the program made a difference in your experience with arthritis? (Yes, No) a) If yes, how? If no, why not? b) Are there things that you can do now that you couldn’t or didn’t do before? c) Has your participation in the program made a difference in: i) the way you feel about your arthritis? How? ii) how you feel about your ability to manage your symptoms? How? iii) your mood? How? iv) your relationships?	
3) For each skill (Progressive Muscle Relaxation, Mini Relaxation Practices, Communicating with Others about Pain and Coping, Managing Unhelpful Mood, Activity Pacing, Pleasant Activities, Pleasant Imagery and Other Distraction Techniques, Problem Solving): i) On a scale of 0–10, with 0 being not helpful at all and 10 being very helpful, how helpful was this skill for you? ii) Are you currently using this skill: (1) Never (2) Occasionally (3) Frequently iii) Is there anything else you would like to add about your use of this skill in terms of things that get in the way of you using it or maybe things that have helped you to use this skill in your daily life?	

### Data analysis

Descriptive statistics were calculated for participant baseline characteristics using means and standard deviations for continuous variables and frequencies and percentages for categorical variables. To analyze participant feedback on the intervention, we used a sequential process informed by the Priority-Sequence Model [21]. We began by calculating means and standard deviations for quantitative survey items to gain a general understanding of participants’ experiences with the intervention. We then examined the open-ended questionnaire items (see Table 2) to further explore participants’ experiences in their own words. The mixed methods approach allowed for added depth in understanding the acceptability of the intervention among participants in this sample. The quantitative methods were primarily used to gauge the helpfulness of the program and skills, and the qualitative methods allowed us to further explore participants’ perceived benefits of the Pain CST intervention.

### Quantitative analysis

For items involving 0–10 scales (Table 2, Questions 1 and 3i) where participants rated the program and individual skills, we calculated means and standard deviations. For categorical items (Questions 2 and 3ii) where participants chose a response from a list, we calculated proportions of participants who reported each response. We also examined responses to these questions based on the participant characteristics of site and gender; for continuous patient characteristics (WOMAC Pain Subscale, Pain Catastrophizing Scale), we divided participants into two groups using a median split. To test for group differences in responses in feedback questions, we conducted t-tests for the 0–10 scales and Chi-Square tests for the categorical items. Analyses were conducted using SAS Version 9.4 (SAS Institute, Cary, NC).

### Qualitative analysis

To analyze open-ended responses to feedback questionnaire items, two researchers (CJD, IG) conducted thematic analysis [22] to further understand participant experiences with the overall program and specific pain coping skills. The researchers conducting the analysis were research assistants, one with extensive experience in qualitative methods and analysis. They were not involved with study development or data collection and did not interact with participants.

To begin the analysis, we first read through a subset of 20 open-ended response entries multiple times while making notes of initial impressions. Each researcher then developed codes related to topics discussed by participants in their open-ended responses. Next, we came together to review the independently-created codes and revised and merged codes to create a codebook. The final codebook (which included ten codes) was created through an iterative process involving collaborative review of the initial codes and questionnaire data to calibrate researchers’ coding process and ensure credibility of the codes. Each researcher then coded half of the open-ended response data using the final codebook. We then met again to review the data organized by code across all open-ended responses. Codes were then collated into broader themes, which captured key elements of participant experiences that were evident across the dataset. Theme development was also an iterative process involving collaborative review among the researchers to ensure themes were clearly named and defined, covered the range of participant responses, and appropriately represented the data. Since responses could contain multiple codes, it was possible for the responses to be categorized under multiple themes. Data was coded using the online Dedoose program 7.0.23 (SocioCultural Research Consultants, LLC; Los Angeles, CA).

## Results

### Participant characteristics

Detailed eligibility, refusal, and enrollment numbers have been published previously [14]. Among 1547 patients who were mailed a letter or self-referred to the study, 308 (20%) screened eligible; of these, 248 (81%) were enrolled and randomized, 2 (< 1%) were subsequently found to be ineligible and 58 (18%) refused / withdrew. Out of the 124 participants in the CST Group, 110 (88.7%) completed 3-month follow-up assessments (Excluded = 3, Withdrew = 3, Missed or Lost to Follow Up = 8). The intervention feedback questions were asked during a separate phone call to preserve blinding of research team members who conducted other assessments at this time point, as well as later follow-up assessments. Of the 110 who completed 3-month assessments, 17 (15.5%) could not be reached for this second phone call. Participant characteristics were similar between these 17 participants and the 93 who did complete feedback questions (see Supplemental Table). One exception was that the mean Pain Catastrophizing scale score was higher for those not completing the feedback questions.

Table 3 shows demographic and clinical characteristics of the 93 participants who completed feedback questions, overall and by study site. Participant characteristics did not differ significantly for participants whose responses were not included for Question 3, as described above. The mean age of participants was 59.4 (SD = 10.2), with 47 (50.5%) identifying as female. A majority of the participants were educated beyond high-school and self-reported having at least enough income to meet their needs. Less than half of the participants reported currently working, and less than half were currently married or living with a partner. When we compared satisfaction ratings against the baseline patient characteristics, we found no significant difference in results between groups.

**Table 3:** Baseline Patient Characteristics^a^

Characteristic	Total (***N*** = 93)	DVAHCS (***N*** = 48, 52%)	UNC (***N*** = 45, 48%)
Female; N (%)	47 (50.5%)	11 (22.9%)	36 (80.0%)
Hispanic/Latino; N (%)	2 (2.2%)	1 (2.1%)	1 (2.2%)
Education - Some education above High School; N (%)	71 (76.3%)	36 (75.0%)	35 (77.8%)
Married or living with partner; N (%)	37 (39.8%)	24 (50.0%)	13 (28.9%)
Working; N (%)	30 (32.3%)	14 (29.2%)	16 (35.6%)
Household financial state: At least enough to meet basic expenses with a little left over; N (%)	58 (62.4%)	31 (64.6%)	27 (60.0%)
Age at baseline, years; mean (SD)	59.4 (10.2)	58.6 (9.3)	60.2 (11.1)
BMI; mean (SD)	36.2 (8.8)	33.3 (6.6)	39.4 (9.8)
WOMAC Total (Scale 0 to 96); mean (SD)	51.4 (19.0)	54.2 (18.2)	48.3 (19.7)
Coping Strategies Questionnaire: Total Coping Attempts (Scale 0 to 216); mean (SD)	95.4 (35.4)	94.1 (39.9)	96.9 (30.1)
Pain Catastrophizing: Total (Scale 0 to 52); mean (SD)	18.5 (11.9)	19.9 (12.4)	17.0 (11.2)
Total Number Comorbidities; mean (SD)	7.9 (3.6)	7.3 (3.5)	8.6 (3.6)

^a^Descriptive statistics were calculated for participant baseline characteristics using means and standard deviations for continuous variables and frequencies and percentages for categorical variables

### Quantitative analysis results

On a scale from 0 to 10, the mean rating for how much the STAART program helped with arthritis symptom management was 8.0 (SD = 2.2) (Table 4). When asked if the program made a difference in their experience with arthritis, 69 (83.1%) participants answered yes. For ratings of helpfulness of specific skills, mean scores (on scales of 0–10) ranged from 7.7 to 8.8. The highest rated skills were Mini Relaxation Practices, Activity Pacing, and Pleasant Activities. Proportions of participants indicating that they used a skill “frequently” ranged from 36.1 to 63.9%. The skills rated as most frequently used included Mini Relaxation Practices, Activity Pacing, and Progressive Muscle Relaxation.

**Table 4:** Program Helpfulness Survey Results^a^

**Question**				
Q1. Scale of 0–10, How much did this program help you manage your arthritis symptoms?; mean (SD)			8.0 (2.2)	
Q2a. Has participating in the program made a difference in your experience with arthritis? (%)			**Yes** 83.1	**No** 16.9
Q3a. Scale of 0–10, How helpful was this skill for you?; mean (SD)				
Progressive Muscle Relaxation			8.1 (2.5)	
Mini Relaxation Practices			8.7 (1.9)	
Communicating with Others about Pain and Coping			7.7 (2.8)	
Managing Unhelpful Mood			7.8 (2.6)	
Activity Pacing			8.5 (2.3)	
Pleasant Activities			8.4 (2.1)	
Pleasant Imagery and Other Distraction Techniques			8.2 (2.5)	
Problem Solving			8.3 (2.4)	
Q3b. Are you currently using this skill? (%)	**Never**	**Occasionally**	**Frequently**	**No Response**
Progressive Muscle Relaxation	12.1	41.0	47.0	0
Mini Relaxation Practices	3.6	37.4	54.2	4.8
Communicating with Others about Pain and Coping	15.7	39.8	36.1	8.4
Managing Unhelpful Mood	12.1	31.3	45.8	10.8
Activity Pacing	6.0	20.5	63.9	9.6
Pleasant Activities	6.0	41.0	41.0	12.1
Pleasant Imagery and Other Distraction Techniques	8.4	31.3	45.8	14.5
Problem Solving	8.4	34.9	42.2	14.5

^a^Descriptive statistics were calculated for survey results using means and standard deviations for continuous variables and frequencies and percentages for categorical variables. Percentages may not total 100 due to rounding

### Qualitative analysis results

We identified themes that were common throughout the dataset in order to organize and understand the participants’ open-ended responses. Four themes identified in the data were: Improved Pain Coping, Mood and Emotional Benefits, Improved Physical Functioning, and Intervention Delivery.

### Improved pain coping

Not surprisingly, given the focus of CST intervention, a common theme was related to participants’ perception of improved ability to cope with arthritis pain. A large portion of participants highlighted the use of new strategies to manage their symptoms. One participant reported, “prior to the program, the pain would change me. I would go to words and get frustrated. But now … I will say climbing the stairway has gotten better. Breathing techniques have helped me to get through that daily.” Utilizing coping strategies taught in the STAART program has reduced this participant’s frustration and allowed for greater mobility. While many participants focused on self-coping strategies, a small subset identified shifts in how they felt about seeking support for coping with pain. One participant stated, “I feel less guilty asking for help.” The CST intervention allowed them to reframe their negative emotions tied to asking others for help and more freely employ support-seeking, which was encouraged in the pain CST intervention. A few participants also noted shifts in their outlook with regards to symptom management. One participant stated “I [no longer] look at it as something that is going to get me down. I do have arthritis, but I can live with it.” This more positive outlook shows an increased confidence in coping with OA.

### Mood and emotional benefits

Another theme focused on various mood and emotional benefits. Many participants highlighted how using skills from the STAART study helped them manage negative emotions related to their pain. One participant noted, “when I first start hurting, I get anxious. Being able to relax is super helpful.” Another participant said, “[these skills] help me to feel relaxed. I feel positive, I don’t feel negative. Just being able to relax helps clear your mind so you’re not feeling anxious.” One participant stated that they were “no longer very depressed and no longer taking depression meds since enrolling in the study.” Participants also highlighted the use of pain coping skills when dealing with other stressful life events. For instance, one participant stated “Last month it really was useful when I was grieving the loss of a family member. I used pleasant imagery to cope.” Another participant noted “It’s very difficult to take my mind off of some of the medical trauma I’ve experienced. The [progressive muscle relaxation skill] has been really helpful with that.” These participants demonstrate that the value of pain coping skills extends beyond pain management.

### Improved physical functioning

This theme encompasses responses in which participants attributed increased physical functioning to the pain CST program. A majority of responses within this theme highlighted improved mobility. For instance, a participant stated, “I feel I can get around easier now.” Another stated more specifically, “I am able to go up and down more stairs now.” Skills taught in the pain CST intervention allowed these participants to build confidence in their mobility. Similarly, a large number of participants highlighted other aspects of improved physical functioning. One participant noted, “it’s easier to bend and pick things up.” Another stated, “I used to wake up feeling stiff and I feel like I don’t as much anymore.” The CST intervention helped these participants make general improvements in their daily functioning and mobility. An increase in physical ability was also highlighted in a small portion of the responses. For instance, one participant shared, “I can go dancing now and ride a bike.” The CST intervention has allowed these individuals to participate in new activities.

### Intervention delivery

Alongside responses relating to personal experiences and perceived benefits, participants also gave feedback regarding aspects of the pain CST program itself. A large portion of these responses were complimentary of the counselors. For instance, one participant stated, “I really looked forward to having these sessions and talking with the counselor. She was so empathetic and understanding and her delivery was great.” The attitude and composure of the counselor seemed to make an impact on many participants’ experiences. Some participants also commented on the method of content delivery. They reported the sessions felt more personal over the phone and expressed doubt about the accessibility of computer-based sessions. One participant pointed out that “not everyone is computer savvy” and another expressed similar concerns, stating, “I don’t have a computer and I don’t know how to work them.” Some participants also highlighted the ease of use of the skills presented in the intervention. The intervention’s accessibility was important to participants and was noted in a positive manner.

## Discussion

Findings from these secondary analyses of the STAART study indicate participants had positive perceptions of the pain CST program. Participants reported that they were very satisfied with the program and that it had a positive impact on their experience with OA. Participants rated all of the pain coping skills as helpful, with Mini Relaxation Practices being rated as the most helpful and Activity Pacing being rated as the most frequently used. For almost all of the skills, over 40% of participants reported frequent use, showing flexibility and breadth in participants’ use of coping strategies. Given the lack of prior studies of behavioral pain interventions among African Americans, these findings of high satisfaction and frequent use of skills from the pain CST program are important and suggest there may be potential for this delivery approach and intervention among African Americans with OA.

Using a mixed methods approach allowed us to go beyond the quantitative indicators of the program’s acceptability and gain a more detailed picture of how the CST intervention affected participants’ experience of OA. The qualitative analysis highlighted program benefits that may have been the underlying factors contributing to participants’ self-reported satisfaction with the intervention. Within participants’ open-ended responses about the program, we identified four themes that provided insight about patients’ experiences with the program: Improved Pain Coping, Mood and Emotional Benefits, Improved Physical Functioning, and the importance of certain aspects of the Intervention Delivery. Since the intervention was focused on pain coping skills, it was no surprise that one of the themes we identified was Improved Pain Coping. Participants’ responses reflected active use of skills taught within the intervention and a growing confidence in ability to apply the skills to manage OA-related symptoms. This is consistent with prior literature on arthritis self-efficacy and its role in the efficacy of self-management interventions [23]. Many participants noted the use of the Activity Pacing skill to delay the onset of pain symptoms, the use of Mini Relaxation to actively combat pain symptoms, and the use of Pleasant Imagery to utilize positive thoughts to lessen their pain experiences. These qualitative results reflected the quantitative data as these 3 skills were the most commonly used by participants. Also within the theme of Improved Pain Coping, some participants made comments that reflect growing pain acceptance, which is important for improving multiple pain-related outcomes [24, 25]; this may lend support to the growing emphasis of incorporating acceptance-based methods into pain CST.

The presence of Mood and Emotional Benefits and Improved Physical Functioning themes was noteworthy because none of the skills taught in the pain CST intervention focused specifically on these themes. With respect to the Mood and Emotional Benefits theme, one potential explanation is that participants used skills taught in the program to combat non-OA stressors. Prior literature has illustrated close associations between emotion, emotion regulation and pain, with evidence for a bidirectional relationship [26, 27]. Given the close associations between emotion and pain-related outcomes, incorporation of emotion regulation strategies within pain CST programs may be beneficial [28]. Participants’ comments on Improved Physical Function are also noteworthy, given that the expected benefits of pain CST typically center on psychological constructs. The frequent use of Activity Pacing (Table 4) helps explain improvement in physical function, as this skill can help patients to accomplish more daily activities through activity-rest cycling. Both clinicians and patients should be informed about potential benefits of pain CST surrounding physical function.

The fourth theme focused on Intervention Delivery. The majority of the responses within this theme praised the counselors for their empathetic and open approach to the participants. Participants often noted the importance of having a friendly counselor, highlighting the personal nature of the pain CST intervention, as well as the need for effectively trained counselors. Many responses within this theme focused on the delivery method of the program, especially the preference of phone contact over in-person appointments. Similarly, many participants stated preferences for phone contact over computer-based programs as they felt they were not skilled enough to utilize a computer for this purpose or they simply did not own one. Recent studies have shown that internet-based pain CST interventions can improve key pain-related outcomes among patients with OA [29, 30]. However, patient preference should be considered with regards to the delivery method of pain CST interventions.

It is also important to note the variability in participants’ ratings and perceptions of the pain CST program. A small number of participants rated both the program overall and several skills as a “0″. Similarly, for a few skills, 12–15% of participants indicated they “never” used the skill. These results show that although overall satisfaction ratings were high, the viewpoint was not uniform. There is still a need to better understand sources of variability in patients’ satisfaction with these types of programs. This information can help improve pain CST programs by focusing efforts on engaging specific groups of patients who may experience more positive outcomes.

This is one of few studies focusing on feedback from a cognitive-behavioral pain intervention. While other research has found that similar cognitive behavioral interventions for pain are effective when adapted for specific populations [31], participant feedback is rarely reported. Another strength of this study is the high proportion of male participants, with a roughly 50% representation, as many OA studies have a majority of female participants. However, one limitation of this study is that many of this study’s male participants were patients from the Department of Veterans Affairs Healthcare system [32], which may limit generalizability. A second limitation is that study participants tended to have relatively high levels of chronic illness, as shown in Table 4. This may also limit external generalizability. A third limitation is the number of participants who did not complete the feedback questions (25%). Although completers vs. non-completers of the feedback questions were similar across most characteristics, non-completers had higher baseline scores on the Pain Catastrophizing Scale. Therefore, results may be biased toward the experience of participants with more favorable pain coping profiles at study initiation. Fourth, although most feedback questionnaires were completed by the study coordinator, some were completed by the CST counselor for logistical reasons; this may have introduced led to more favorable responses regarding intervention satisfaction, due to social desirability. Finally, as this is a culturally enhanced Pain CST model for African Americans with OA, the results may not be generalizable to participants from other racial backgrounds.

## Conclusions

The high ratings and positive responses present in this secondary analysis support the overall acceptability of the culturally enhanced pain CST program among African Americans with OA. The broad array of impacts noted by participants, from improved coping to improved physical functioning, are particularly noteworthy. Pain CST is not widely available or familiar as a component of OA management, but these findings point toward the need to increase its uptake, particularly among those who may benefit most through remotely delivered interventions. We recommend efforts to increase access to pain CST programs, as well as identification of strategies to teach these skills to patients within team-based primary care environments.

## Supplementary information

**Additional file 1: Table S1.** Feedback Completers vs Non-completers Baseline Characteristics^a^.

## Data Availability

The datasets generated during the current study are not publicly available but are available (deidentified) from the corresponding author on reasonable request.

## Abbreviations

CST: Coping Skills Training
OA: Osteoarthritis
STAART: Pain Coping Skills Training for African Americans with Osteoarthritis
UNC: University of North Carolina
DVAHCS: Durham Veteran Healthcare System Healthcare System
WOMAC: Western Ontario and McMaster Universities Osteoarthritis Index
