# Supplementary material for: Acceptability of telephone-based pain coping skills training among African Americans with osteoarthritis enrolled in a randomized controlled trial: a mixed methods analysis
Source: BMC Musculoskelet Disord. 2020 Aug 14;21:545. doi: 10.1186/s12891-020-03578-7 (PMC7427940; doi:10.1186/s12891-020-03578-7)
Supplement: Supplementary file 1 — Additional file 1: Table S1. Feedback Completers vs Non-completers Baseline Characteristicsa. [file 12891_2020_3578_MOESM1_ESM.docx]

Supplemental Table 1. Feedback Completers vs Non-completers Baseline Characteristics^a^

| **Characteristic** | **Feedback Completers**  **(N=93)** | **Non-Completers**  **(N=31)** | **p value** |
| --- | --- | --- | --- |
| Female; N (%) | 47 (50.5%) | 14 (45.2%) | 0.60 |
| Hispanic; N (%) | 2 (2.2%) | 1 (3.3%) | 0.74 |
| Education - Some education above High School; N (%) | 71 (76.3%) | 21 (67.7%) | 0.34 |
| Married or living with partner; N (%) | 37 (39.8%) | 14 (45.2%) | 0.60 |
| Working; N (%) | 30 (32.3%) | 13 (41.9%) | 0.33 |
| Household financial state: At least enough to meet basic expenses with a little left over; N (%) | 58 (62.4%) | 18 (58.1%) | 0.67 |
| Age at baseline, years; mean (SD) | 59.4 (10.2) | 58.5 (8.6) | 0.64 |
| BMI; mean (SD) | 36.2 (8.8) | 33.7 (7.0) | 0.16 |
| WOMAC Total (Scale 0 to 96); mean (SD) | 51.4 (19.0) | 57.9 (19.0) | 0.10 |
| Coping Strategies Questionnaire: Total Coping Attempts (Scale 0 to 216); mean (SD) | 106.1 (36.6) | 100.0 (36.3) | 0.42 |
| Pain Catastrophizing: Total (Scale 0 to 52); mean (SD) | 18.5 (11.9) | 24.3 (14.5) | 0.03 |
| Total Number Comorbidities; mean (SD) | 7.9 (3.6) | 8.9 (4.7) | 0.21 |

**^a^**Descriptive statistics were calculated for participant baseline characteristics using means and standard deviations for continuous variables and frequencies and percentages for categorical variables.
